# Use of a Parasitic Wasp as a Biosensor

**DOI:** 10.3390/bios4020150

**Published:** 2014-05-08

**Authors:** Dawn Olson, Glen Rains

**Affiliations:** 1 Crop Protection and Management Research Unit, Agricultural Research Service, United States Department of Agriculture, P.O. Box 748, Tifton, GA 31793, USA; 2 Department of Entomology, University of Georgia, Tifton, GA 31793, USA; E-Mail: grains@uga.edu

**Keywords:** *Microplitis croceipes*, odor concentration, associative learning, habituation

## Abstract

Screening cargo for illicit substances is in need of rapid high-throughput inspection systems that accurately identify suspicious cargo. Here we investigate the ability of a parasitic wasp, *Microplitis croceipes* to detect and respond to methyl benzoate, the volatile component of cocaine, by examining their response to training concentrations, their sensitivity at low concentrations, and their ability to detect methyl benzoate when two concealment substances (green tea and ground coffee) are added to the testing arena. Utilizing classical associative learning techniques with sucrose as reward, we found that *M. croceipes* learns individual concentrations of methyl benzoate, and they can generalize this learning to concentrations 100× lower than the training concentration. Their sensitivity to methyl benzoate is very low at an estimated 3 ppb. They are also able to detect methyl benzoate when covered completely by green tea, but were not able to detect methyl benzoate when covered completely by coffee grounds. Habituation to the tea and coffee odors prior to testing improves their responses, resulting in effective detection of methyl benzoate covered by the coffee grounds. With the aid of the portable device called ‘the wasp hound’, the wasps appear to have potential to be effective on-site biosensors for the detection of cocaine.

## 1. Introduction

Screening cargo for illicit substances has been time constrained, in part due to false positive responses, so that high-throughput inspection systems that rapidly screens and accurately identifies suspicious cargo are still required [1]. Chemical detection requires that the substance has volatile properties and for cocaine detection, the volatile component is methyl benzoate [2,3,4]. The two trace detection systems that are most commonly employed at US checkpoints are canines and ion mobility spectrometry (IMS) [1]. Although generally reliable, canines can only work limited hours, cannot be used when ill, are labor-intensive to train, and are subject to handler bias, whereas, IMS must be close to the surface of suspected area which may necessitate unloading the cargo [1]. More recently, Lai *et al.* [1] showed that solid phase microextraction (SPME) coupled to IMS was effective at detecting methyl benzoate, but some interference compounds produced false positives so that higher levels of specificity of SPME are being investigated. Therefore, there remains a need for sensitive and accurate means of identifying suspicious cargo.

*Microplitis croceipes* (Cresson) (Hymenoptera: Braconidae), a specialized parasitic wasp of the larvae of their highly polyphagous hosts, *Helicoverpa zea*, *Heliothis virescens* and *Heliothis armigera* (Lepidoptera: Noctuidae), has been used as a model species for the study of insect learning for more than 25 years. Previous work has shown that *M. croceipes* employs classical Pavlovian conditioning, defined as a temporal pairing of a conditioned stimulus with an unconditioned reward, to learn novel visual [5] as well as a broad range of chemical cues [6,7,8,9,10,11,12]. They have been shown to differentiate conditioned odors from closely related chemicals on the basis of molecule chain length and type and position of functional groups [11]. Following conditioning to a blend of odors, they subsequently respond to the full blend, as well as to combinations of the individual chemicals [13] and they are able to detect individual compounds when imbedded in a 1:1:1 mixture of closely related compounds [8]. This wasp’s learning is also concentration dependent [9] as has been found in the honey bee [14,15]. Learning generalization is defined as responding to one stimulus as a result of training involving some other, usually similar stimulus [16]. *Microplitis croceipes*’ concentration learning is also chemical dependent, and they can show no learning generalization (respond to the trained concentration only), learning generalization to the training concentration as well as to either higher or lower than the training concentration, or to both higher and lower than the training concentration [9]. The use of negative training experience (odor plus water), and/or additional positive training experience (odor plus sucrose) allowed modification of their responses so that a desired range of concentrations of a targeted compound could be detected [9]. 

Utilizing living insects as biosensors requires that they are contained. For this reason, a portable device called the ‘wasp hound’ was developed [17]. Multiple trained individuals can be utilized in the ‘wasp hound’ which reduces false positives by averaging responses [8]. The wasps walk freely within a cartridge inserted into the ‘wasp hound’ container and respond to the sample air with specific foraging behaviors [10]. A video camera integrated in the ‘wasp hound’ captures their behavior and a Windows-based behavioral analysis program graphs and identifies the wasp’s response [17]. The detection response is obtained in 10–20 s. The chemical concentration conditioned wasps and the Wasp Hound can be transported to specific areas where the interest lies in the detection of targeted odors. This methodology is of particular interest in the early detection of plant disease, food contamination, pest infestations, cadavers, defense related chemicals, and illicit substances.

The primary aim of this study is to investigate the ability of *M. croceipes* to detect and respond to methyl benzoate. The first objective is to examine the wasp’s response to training concentrations and higher and lower concentrations of methyl benzoate. The second objective is to examine their sensitivity to methyl benzoate by training and testing them to extremely low concentrations. The third objective is to examine their ability to detect methyl benzoate when two concealment substances (green tea and ground coffee) are added to the testing arena.

## 2. Experimental Section

The larval parasitoid, *Microplitis croceipes* (Cresson) (Hymenoptera: Braconidae) were reared on corn ear worm, *Helicoverpa zea* (Boddie) (Lepidoptera: Noctuidae) larvae according to the method described by Lewis and Burton [18]. Adult wasps were maintained in a Plexiglas cage (30 × 30 × 17 cm) within an environmental chamber with a temperature of 28 °C, 50–70% RH and 16L:8D photocycle. The wasps were provided with water only for 48 h prior to the experiments. Female and male wasps that were 3 days old, with females mated and without oviposition experience at the time of the odor conditioning were used in the experiments. Males are less abundant and have lower longevity than females so when males were used, equal numbers were placed into each treatment. All conditioning and testing protocols were conducted at 23 ± 2 °C under a fume hood. At the time of these experiments, the wasp hound was being upgraded. Therefore, we tested wasp responses with table top bioassays instead of the wasp hound as responses under both methods result in similar conclusions [8,19,20,21].

### 2.1. Conditioning and Testing of Response to Concentrations of Methyl Benzoate

#### 2.1.1. Conditioning

Food-deprived wasps were conditioned to odor concentration by providing them with a positive feeding experience (presentation of sucrose-water in association with the odor concentration) to 30 μL of the methyl benzoate training solution dissolved in dichloromethane (DCM) at 10 μg/mL, 100 μg/mL or 1000 μg/mL. A solution of 1000 μg/mL was prepared and serially diluted to obtain the various concentrations. Concentrations of methyl benzoate were pipetted onto a filter paper (2.5cm·d) and the solvent was allowed to evaporate for one min before placing the filter paper in the center of an open glass Petri dish (10 cm·d and 1 cm·h). A conditioning arena was created using a 9 cm·d, 1.5 cm·h metal ring covered with aluminum foil. This arena was subsequently placed inside the Petri dish covering the odor source. A small (4 mm^2^) piece of filter paper laden with a 1 molar sucrose solution was placed at the center of the aluminum foil. A total of 9 holes (1mm·d each) were punctured around the tissue paper so that parasitoids would be exposed to the diffusing volatiles while feeding on the sugar water. Parasitoids were introduced into the arena from a glass drum vial and allowed to feed 10 s before being removed. This acquisition trial was repeated three times with 30 s interval between feedings. All training and testing was done under a chemical fume hood. The conditioning arena, the odor source and the sugar water were renewed every 10 min. Methyl benzoate was obtained from Sigma-Aldrich (>98% purity). A total of 60 wasps were trained to 10 μg/mL, 60 wasps were trained to 100 μg/mL and 80 wasps were trained to 1000 μg/mL.

#### 2.1.2. Test

We recorded a food-specific conditioned behavioral complex based on a modification of an ‘area restricted search’ [22]. This response complex includes intense substrate antennation with frequent tight turning at an odor portal (s), a distinct lowering of the head contacting the substrate around the odor portal(s) with the proboscis, as well as attempts to enter the odor portal(s) [8]. Response measurements recorded utilizing the same type of arena as in conditioning was the wasp’s total tenure time and number of ≥45° turns at the odor portal. The odor portal consisted of 3 1mm d holes punctured in the center of the aluminum foil and spaced 1 mm apart. The bioassay ended when the wasps stopped their food-specific behaviors and walked or flew away. Wasps were tested to the methyl benzoate concentration they had associated with the feeding experience and to concentrations 10× and 100× lower and 10× higher than the training concentration. Of the 60 wasps trained to 10 μg/mL, 20 were tested to 10 μg/mL (control), 20 were tested to 1 μg/mL and 20 were tested to 100 μg/mL. Of the 60 wasps trained to 100 μg/mL, 20 were tested to 100 μg/mL (control), 20 were tested to 10 μg/mL and 20 were tested to 1000 μg/mL. Of the 80 wasps trained to 1000 μg/mL 20 wasps were tested to 1000 μg/mL, 20 wasps to 100 μg/mL, 20 wasps to 10 μg/mL, and 20 wasps to 1μg/mL. Training and testing spanned 4 days with 5 wasps trained and tested under each treatment each day.

### 2.2. Sensitivity Tests

#### 2.2.1. Conditioning

A total of 10 wasps for each concentration were trained as above to 0.001 μg/mL, 0.01 μg/mL, 0.1 μg/mL or 1 μg/mL of methyl benzoate solution dissolved in DCM. 

#### 2.2.2. Test

Wasp responses to the 0.001 μg/mL, 0.01 μg/mL, 0.1 μg/mL or 1 μg/mL methyl benzoate concentrations they had associated with the feeding experience were recorded as above. A total of 10 wasps were tested to the concentration utilized in training.

### 2.3. Response to Methyl Benzoate with Green Tea or Ground Coffee

#### 2.3.1. Conditioning

A total of 38 wasps were trained as above to 1000 μg/mL of methyl benzoate solution dissolved in DCM. After training, 19 of the wasps were placed in a plexiglass cage with 3 g of either green tea (from China) or coffee grounds (Folgers, dark roast) evenly spread on the bottom of the cage and left for 30 min. prior to testing to habituate them to the respective odors. 

#### 2.3.2. Test

Wasp responses to the 1000 μg/mL methyl benzoate with green tea or ground coffee were recorded as above. One gram of green tea or ground coffee was evenly distributed in the Petri dish so that the filter paper with the compound was 100% covered (no filter paper was visible) as well as an area of 2 cm surrounding the filter paper. The depth of the leafy green tea was 3 mm, whereas the depth of the ground coffee was 1 mm. A total 38 wasps were tested to the training concentration with 19 of these being wasps that had not been habituated to the green tea or coffee and 19 wasps that had been habituated to the green tea or coffee. The experiments spanned 4 days with 4–5 wasps trained and tested each day.

#### 2.3.3. Conditioning

A total of 24 wasps were trained as above to 1000 μg/mL of methyl benzoate solution dissolved in DCM. After training, 12 of the wasps were placed in a plexiglass cage with 3 g of either green tea or coffee grounds as described above.

#### 2.3.4. Test

Wasp responses to the 1000 μg/mL methyl benzoate with green tea or ground coffee were recorded as above. One gram of green tea or ground coffee was evenly distributed in the Petri dish so that the filter paper with the compound was 70% covered (30% of the filter paper visible) as well as an area of 2 cm surrounding the filter paper. The depth of the green tea and ground coffee was as described above. A total of 24 wasps were tested to the training concentration with 12 of these being wasps that had not been habituated to the green tea or coffee and 12 wasps that had been habituated to the green tea or coffee. The experiments spanned 2 days with 6 wasps trained and tested each day.

### 2.4. Statistics

ANOVA was used to test the effect of training concentration on wasp responses to the 10× lower, 100× lower, 1000× lower and the 10× higher concentrations with Tukey’s HSD used to separate the means [23]. To meet model assumptions of equal variance and normal distribution, tenure time and number of turns for wasps trained to 10 μg/mL and 1000 µg/mL were sqrt-transformed and those trained to 100 μg/mL were ln-transformed. For the sensitivity test, tenure time was sqrt-transformed and the number of turns was ln-transformed. For the habituation test involving coffee, the number of turns and tenure time were sqrt-transformed. 

## 3. Results and Discussion

### 3.1. Response to Concentrations of Conditioned Odors

**Figure 1:**
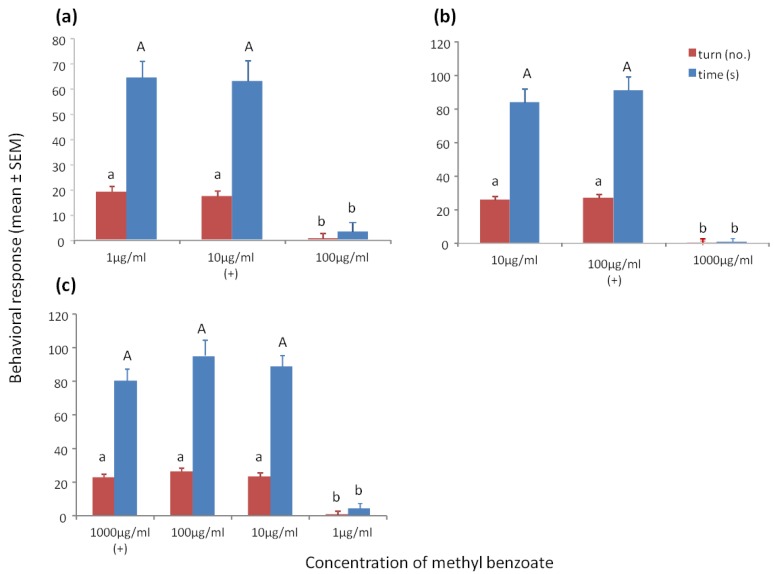
(**a**) Mean ° SEM tenure time (s) and number of ≥45° turns at the odor portal of wasps trained to 10 μg/mL methyl benzoate and tested to methyl benzoate at 1 μg/mL, 10 μg/mL and 100 μg/mL. (**b**) Mean ° SEM tenure time and number of turns at the odor portal of wasps trained to 100 μg/mL methyl benzoate and tested to methyl benzoate at 10 μg/mL, 100 μg/mL, and 1000 μg/mL. (**c**) Mean ° SEM tenure time and number of turns at the odor portal of wasps trained to 1000 μg/mL methyl benzoate and tested to methyl benzoate at 1 μg/mL, 10 μg/mL, 100 μg/mL and 1000 µg/mL. N = 20. (+) = presented a positive feeding experience in association with the chemical concentration. Means with different letters above the bars indicate significant difference at alpha 0.05 using Tukey’s HSD for both turns (lower case) and time (upper case).

Wasps trained to 10 μg/mL methyl benzoate and tested to methyl benzoate at 1 μg/mL, 10 μg/mL and 100 μg/mL showed a significant increase in turning (F_2/59_ = 88.98, *p* < 0.001) and time spent at the odor portal (F_2/59_ = 79.79, *p* < 0.001) of the training concentration and the 10× lower concentration of 1 μg/mL, but not to the 10× higher concentration of 100 μg/mL (Figure 1a). 

Wasps trained to methyl benzoate at 100 μg/mL, and tested to 10 μg/mL, 100 μg/mL and 1000 μg/mL, showed a significant increase in turning (F_2/59_ = 521.78, *p* < 0.001) and time spent at the odor portal (F_2/59_ = 370.35, *p* < 0.001) of the training concentration and the 10× lower concentration of 10 μg/mL, but not to the 10× higher concentration of 1000 μg/mL (Figure 1b). 

Wasps trained to methyl benzoate at 1000 μg/mL, and tested to 1 μg/mL, 10 μg/mL, 100 μg/mL, and 1000 μg/mL showed a significant increase in turning (F_3/79_ = 93.33, *p* < 0.001) or time spent at the odor portal (F_3/79_ = 87.73, *p* < 0.001) of the training concentration and the 10× and 100× lower concentrations, but not the 1000× lower concentration (Figure 1c). To obtain positive responses to all concentrations between 0.01 µg/mL and 1000 µg/mL, an additional and similar positive exposure to sucrose at 1 µg/mL would be needed [9]. 

### 3.2. Sensitivity Tests

**Figure 2:**
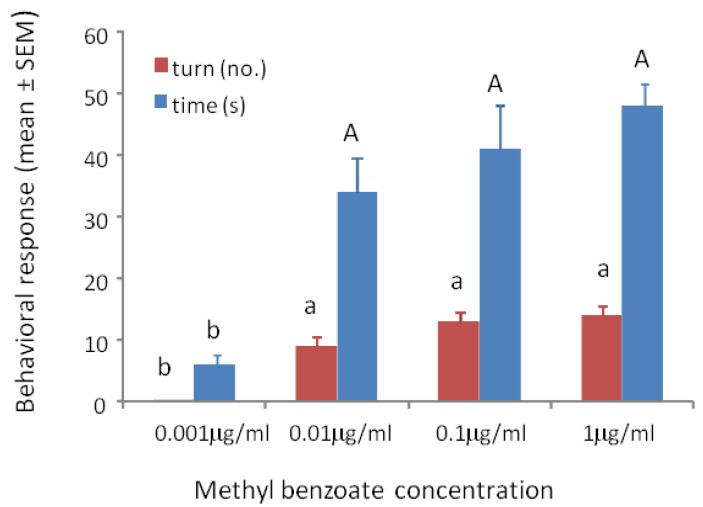
Mean ° SEM tenure time (s) and number of ≥45° turns at the odor portal of wasps trained and tested to 0.001 μg/mL, 0.01 μg/mL 0.1 μg/mL and 1 μg/mL methyl benzoate. N = 10. Means with different letters above the bars indicate significant difference at alpha 0.05 using Tukey’s HSD for both turns (lower case) and time (upper case).

Wasps trained to methyl benzoate at 0.001 μg/mL, 0.01 μg/mL, 0.1 μg/mL or 1 μg/mL and tested to their training concentration showed significant differences in turning frequency (F_3/39_ = 81.56, *p* < 0.001) and time spent at the odor portal (F_3/59_ = 20.19, *p* < 0.001). The wasps showed positive responses to all concentrations except the 0.001 μg/mL concentration (Figure 2).

### 3.3. Response to Methyl Benzoate with Green Tea and Ground Coffee

**Figure 3:**
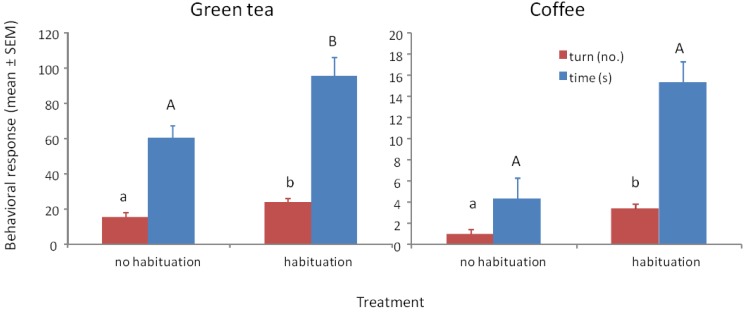
(**a**) Mean ° SEM tenure time (s) and number of ≥45° turns at the odor portal of wasps trained to 1000 μg/mL methyl benzoate and tested to 1000 μg/mL methyl benzoate plus 1 g of green tea with and without a 30 min. exposure to green tea after training (=habituation to tea odor) N = 19. (**b**) Mean ° SEM tenure time and number of turns at the odor portal of wasps trained to 1000 μg/mL methyl benzoate and tested to 1000 μg/mL methyl benzoate plus 1 g of ground coffee after training (=habituation to coffee odor) N = 24. Means with different letters above the bars indicate significant difference at alpha 0.05 using Tukey’s HSD for both turns (lower case) and time (upper case).

Wasps trained to methyl benzoate at 1000 μg/mL and tested to their training concentration with green tea with and without habituation to the tea odor showed significant differences in turning frequency (F_1/37_ = 5.79, *p* = 0.021) and time spent at the odor portal (F_1/37_ = 5.27, *p* = 0.028) (Figure 3a). All of the wasps showed a positive response after habituation, whereas 4 of the 19 (21%) wasps without habituation to the tea odor did not respond and showed some aversion behavior (prone to fly or walk away from odor portal). Although habituation to tea odor improved the responses, 79% of the non-habituated wasps showing a positive response would be highly indicative of the presence of methyl benzoate. When tested to methyl benzoate that is 30% covered with the tea, 100% of non-habituated wasps showed strong positive responses in number of turns (mean ± SEM = 20.7 ± 2.3, N = 13) and time (63.3 ± 7.4 s, N = 13) suggesting that the tea is physically preventing some of the methyl benzoate volatiles from entering the head space.

Wasps trained to methyl benzoate at 1000 μg/mL and tested to their training concentration with ground coffee with and without habituation to the coffee odor showed significant differences in turning frequency (F_1/47_ = 13.66, *p* ≤ 0.001) and time spent at the odor portal (F_1/47_ = 16.48, *p* < 0.001) (Figure 3b). A total of 17 out of 24 (71%) wasps showed a positive response after habituation, whereas 17 of the 24 (71%) wasps without habituation to the coffee odor did not respond and showed some aversion behavior. Habituation to coffee odor appears necessary when the coffee grounds completely cover the filter paper with the methyl benzoate. The 71% of wasps showing a positive response would be highly indicative of the presence of methyl benzoate. When tested to methyl benzoate that is 30% covered with the coffee grounds, 100% of non-habituated wasps showed strong positive responses in number of turns (mean ± SEM = 18.6 ± 2.3, N = 13) and time (71.6 ± 9.4 s, N = 13) suggesting that the coffee grounds are physically preventing some of the methyl benzoate volatiles from entering the headspace.

The results show that *M. croceipes* can effectively learn and respond to methyl benzoate at a range of concentrations. Furthermore, they generalize concentrations of this compound to the training concentration and lower concentrations. To effectively utilize them as a biosensor, the training concentration should be at the highest level of this compound that are typically found in cargo holds so that a range of responses–at the training concentration and those lower–can be obtained.

The wasps’ sensitivity to methyl benzoate is quite high. The volume of the container used in training and testing was 104.55 cm^3^, and with the assumption that all of the methyl benzoate is in the gas phase, the estimated concentration at the lowest level of response (0.01 μg/mL) is 3 parts-per-billion (ppb). However, given that the volatility of methyl benzoate is low (boiling point of 199.6 °C) it is unlikely that the entire compound was in the gas phase given the short duration of the experiments conducted under simple diffusion conditions. Therefore, concentration detection by *M. croceipes* may be even lower than what we have estimated. Canines are able to inhale headspace volatiles and have been reported to detect compounds in the ppt’s [24]. With the aid of the wasp hound that pulls air through the system, *M. croceipes* may further increase their level of detection. The level of sensitively of *M. croceipes* has obvious practical applications for detection of illicit chemical substances or other compounds where information on their presence or absence is of interest.

Despite the physical prevention of some of the methyl benzoate volatiles from entering the head space when tea or coffee totally covers the filter paper, the wasps were still able to detect it and effectively respond. Furthermore, the wasps did not have to be habituated to the tea in order to obtain these positive responses.

A crucial avenue of further investigation is the testing of *M. croceipes*’ ability to detect methyl benzoate, especially at low concentrations, when other often encountered concealment chemicals are also present [1]. *Microplitis croceipes* can detect a trained compound when it is embedded in a 1:1:1 mixture of closely related compounds [8], and concentrations and not the proportions of a compound in a mixture are more relevant to this species (Olson and Wäckers, unpublished data). The ‘Wasp Hound’ employs multiple individuals and air is pulled through the chamber, which increases the probability of detection of the target odor. Furthermore, we have shown here that habituation to tea and coffee significantly increases their detection ability. These attributes have the potential to correct for possible detection interference caused by the presence of other volatile chemical substances. In addition, given that larger concentrations of methyl benzoate than those investigated here may be present, further tests will need to be conducted to determine the highest concentration that the wasps are able to learn and report. 

## 4. Conclusions

Utilizing classical associative learning techniques with sucrose as reward, we found that *M. croceipes* learns individual concentrations of methyl benzoate, and they generalize this learning to the training and lower concentrations. Their sensitivity to methyl benzoate is very low at an estimated 3 ppb. The wasps are also able to detect methyl benzoate when covered completely by green tea, but were less able to detect methyl benzoate when covered completely by coffee grounds. Habituation to the tea and coffee odors prior to testing improves their responses, resulting in effective detection of methyl benzoate covered by the coffee grounds. With the aid of the Wasp Hound portable device, the wasps appear to have potential to be effective on-site biosensors for the detection of cocaine.
